# Novelty, conditioning and attentional bias to sexual rewards

**DOI:** 10.1016/j.jpsychires.2015.10.017

**Published:** 2016-01

**Authors:** Paula Banca, Laurel S. Morris, Simon Mitchell, Neil A. Harrison, Marc N. Potenza, Valerie Voon

**Affiliations:** a Department of Psychiatry, University of Cambridge, Cambridge, UK; b PhD Programme in Experimental Biology and Biomedicine, Center for Neuroscience and Cell Biology, University of Coimbra, Portugal; c Institute for Biomedical Imaging and Life Sciences, University of Coimbra, Portugal; d Behavioral and Clinical Neurosciences Institute, University of Cambridge, Cambridge, UK; e Cambridgeshire and Peterborough NHS Foundation Trust, Cambridge, UK; f Department of Psychiatry, Brighton and Sussex Medical School, Brighton, UK; g Departments of Psychiatry, Neurobiology and Child Study Center, CASA Columbia and Connecticut Mental Health Center, Yale University, New Haven, CT, USA

**Keywords:** Novelty, Cue-conditioning, Sexual reward, Dorsal cingulate habituation, Addiction, Attentional bias

## Abstract

The Internet provides a large source of novel and rewarding stimuli, particularly with respect to sexually explicit materials. Novelty-seeking and cue-conditioning are fundamental processes underlying preference and approach behaviors implicated in disorders of addiction. Here we examine these processes in individuals with compulsive sexual behaviors (CSB), hypothesizing a greater preference for sexual novelty and stimuli conditioned to sexual rewards relative to healthy volunteers. Twenty-two CSB males and forty age-matched male volunteers were tested in two separate behavioral tasks focusing on preferences for novelty and conditioned stimuli. Twenty subjects from each group were also assessed in a third conditioning and extinction task using functional magnetic resonance imaging. CSB was associated with enhanced novelty preference for sexual, as compared to control images, and a generalized preference for cues conditioned to sexual and monetary versus neutral outcomes compared to healthy volunteers. CSB individuals also had greater dorsal cingulate habituation to repeated sexual versus monetary images with the degree of habituation correlating with enhanced preference for sexual novelty. Approach behaviors to sexually conditioned cues dissociable from novelty preference were associated with an early attentional bias to sexual images. This study shows that CSB individuals have a dysfunctional enhanced preference for sexual novelty possibly mediated by greater cingulate habituation along with a generalized enhancement of conditioning to rewards. We further emphasize a dissociable role for cue-conditioning and novelty preference on the early attentional bias for sexual cues. These findings have wider relevance as the Internet provides a broad range of novel and potentially rewarding stimuli.

## Graphical abstract

**Figure:**
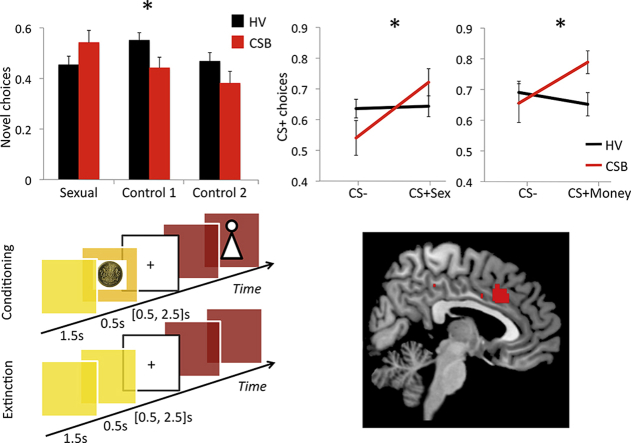

## 1 Introduction

Why is online surfing so compulsively engaging to many individuals? The Internet provides a large source of novel and potentially rewarding stimuli. Novelty-seeking, attentional bias and cue-conditioning are fundamental processes that may drive unconscious preference and approach decisions in daily life. These processes may also contribute to the development and maintenance of disorders of addiction.

Novelty-seeking may be both a predictor and consequence of disorders of addiction. This trait, which is often assessed using the Zuckerman's sensation-seeking scale, has been repeatedly found elevated in diverse behavioral and substance addictions (Belin et al., 2011, Redolat et al., 2009). A suggested explanation for this strong relationship relies on the hypothesis that exposure to novelty may activate, at least in part, the same neural machinery that mediates the rewarding effects of drugs of abuse (Bardo et al., 1996). In rodent studies, novelty preference predicts a transition towards compulsive cocaine-seeking behaviors (Belin and Deroche-Gamonet, 2012). In human studies, sensation-seeking is associated prospectively with binge drinking in adolescents (Conrod et al., 2013).

Conditioned signals or cues in our environment may also significantly influence behavior. The smell of cigarettes, places or friends associated with drug use, or the sight of money may act as conditioned cues and may enhance reactivity and trigger cravings, urges and relapse in disorders of addiction (for review see (Childress et al., 1993)). These cues are neutral stimuli that may inadvertently acquire motivational significance through the process of conditioning with repeated pairing with either drug rewards or other biologically relevant natural rewards such as food (Jansen, 1998) or sex (Pfaus et al., 2001, Toates, 2009).

The processing of novelty and learning has been proposed to include a functional polysynaptic loop involving the hippocampus, ventral striatum, and midbrain dopaminergic region (Lisman and Grace, 2005). Detection of novelty, long-term memory encoding and learning involves dopaminergic activity that enhances hippocampal synaptic plasticity which, through glutamatergic projections to the ventral striatum, relays information to the ventral tegmental area (VTA) which then projects directly back to the hippocampus (Knight, 1996, Lisman and Grace, 2005). With repeated exposure, the hippocampus and midbrain dopaminergic responses to novelty decrease, conferring habituation when stimuli become familiar (Bunzeck and Duzel, 2006, Bunzeck et al., 2013). Converging primate and human studies also show that phasic dopaminergic activity encodes prediction error, a comparison between the actual and expected outcomes indicating an unexpected salient outcome, acting as a teaching signal underlying conditioning processes (Schultz et al., 1997). Mesolimbic dopaminergic cell bodies in the midbrain project to a network including the striatum, dorsal anterior cingulate cortex (dACC) and hippocampus (Williams and Goldman-Rakic, 1998). The dACC is implicated in attentional responding to novel and salient events and in processing reward anticipation and prediction error (Ranganath and Rainer, 2003, Rushworth et al., 2011).

In addition to novelty-seeking and cue-conditioning influences, the tendency to preferentially process cues related to the object of addiction (attentional biases) is also an important feature that characterizes disorders of addiction (Ersche et al., 2010, van Hemel-Ruiter et al., 2013, Wiers et al., 2011). The influence of emotional stimuli on attentional processes is widely reported both in healthy and clinical samples (Yiend, 2010). Attentional biases towards substance-related stimuli have been found in substance-use disorders for alcohol, nicotine, cannabis, opiates and cocaine (Cox et al., 2006). Moreover, a direct association between highly arousing sexual pictures and attentional interference has also been found in healthy individuals, which seems to be influenced by sexuality-related attitudes and sexual motivation (Kagerer et al., 2014, Prause et al., 2008). We have previously extended these findings to individuals with compulsive sexual behaviour (CSB) using a dot-probe task (Mechelmans et al., 2014).

With increasing access to the Internet, there is growing concern regarding the potential for excessive use. A study that assessed the predictive power of several types of Internet applications (gaming, gambling, email, etc.) on the development of compulsive Internet use suggested that online sexually explicit stimuli have the highest potential for addictive/compulsive use (Meerkerk et al., 2006). Online explicit stimuli are vast and expanding, and this feature may promote escalation of use in some individuals. For instance, healthy males viewing repeatedly the same explicit film have been found to habituate to the stimulus and find the explicit stimulus as progressively less sexually arousing, less appetitive and less absorbing (Koukounas and Over, 2000). However, subsequent exposure to a novel explicit film segment increases the levels of sexual arousal and absorption to the same previous levels prior to habituation, suggesting important roles for novelty and habituation. Imaging studies have identified a specific network for the neural processing of sexual stimuli in healthy humans, comprising the hypothalamus, nucleus accumbens, orbitofrontal, occipital and parietal areas (Wehrum et al., 2013, Wehrum-Osinsky et al., 2014). This neural network, which is independent of general emotional arousal, is found both in men and women although men show overall stronger activations than women, which might be indicative of a stronger sexual responsivity in men. The same neural network activates for stimuli conditioned sexual arousal, with a gender effect in the same direction (Klucken et al., 2009).

In our study, we assess novelty, attentional bias and cue-conditioning to online explicit sexual material in individuals with CSB. These processes are highly relevant to substance-use disorders and may also be relevant to CSB. Online sexually explicit stimuli have significant potential for compulsive use, and CSB is relatively common, occurring in 2–4% in community and college-based young adults and in psychiatric inpatients (Grant et al., 2005, Odlaug and Grant, 2010, Odlaug et al., 2013). CSB is associated with significant distress, feelings of shame and psychosocial dysfunction. Although a working group for the 11th edition of the International Classification of Diseases is currently proposing to include CSB as an impulse-control disorder (Grant et al., 2014), CSB was not included in DSM-5, albeit with some controversy (Toussaint and Pitchot, 2013), largely due to limited data. Thus, further studies are needed. Understanding the similarities and differences between CSB and other psychiatric disorders, particularly impulse-control disorders and addictions, may help with classification efforts as well as with the development of improved prevention and treatment approaches.

We have previously found that individuals with CSB demonstrate greater regional brain activation in response to explicit sexual cues in the ventral striatum, dorsal anterior cingulate cortex (dACC) and amygdala, regions implicated in drug cue reactivity and craving in disorders of addiction (Voon et al., 2014). Functional connectivity of this network, and particularly the dACC, was associated with greater sexual desire or motivation to explicit stimuli. We further observed that individuals with CSB, as compared to those without, demonstrate an early attentional bias towards sexually explicit cues (Mechelmans et al., 2014). This early attentional bias was proposed to reflect facilitatory mechanisms underlying the motivational effect of cues conditioned to sexual outcomes. Here, we deepen our research focus investigating the mechanisms underlying the development of enhanced attentional bias and cue reactivity in CSB by assessing both behavioral and neural responses to novelty and to cue-conditioning in response to explicit sexual stimuli.

We conducted two behavioral tasks outside of the scanner to assess choice preference for novel versus familiar sexual stimuli and choice preference for cues conditioned to Sexual, Monetary and Neutral stimuli. We hypothesized that CSB individuals relative to healthy volunteers (HVs) would have greater choice preference to novel relative to familiar images in the Sexual condition but not in the control condition. We further hypothesized that CSB subjects would have greater choice preference to the conditioned cues in the Sexual condition but not in the Monetary condition.

Participants also performed a functional magnetic resonance imaging (fMRI) conditioning and extinction task involving conditioning to Sexual, Monetary and Neutral images. Two neutral stimuli were randomly paired with different sexual images shown repeatedly during conditioning. In the outcome phase of the conditioning arm, neural habituation to the sexual images was assessed by evaluating the change in neural activity of each different sexual image over time focusing on the repeated exposure thereby dissociating the analysis of the conditioning and outcome phases. We hypothesized that CSB subjects relative to HVs would show enhanced neural activity to the Sexual versus Neutral conditioned stimuli particularly in the dACC and striatum, regions previously identified in sexual cue reactivity in CSB subjects (Voon et al., 2014). We further hypothesized that CSB subjects compared to HVs would show greater neural habituation to Sexual compared to Neutral stimuli.

## 2 Method

### 2.1 Recruitment

The recruitment has been extensively described elsewhere (Voon et al., 2014). CSB subjects were recruited via Internet-based advertisements and therapist referrals. HVs were recruited from community-based advertisements in East Anglia. CSB subjects were interviewed by a psychiatrist to confirm they fulfilled diagnostic criteria for CSB (proposed diagnostic criteria for Hypersexual Disorder; criteria for sexual addiction) (Carnes et al., 2001, Kafka, 2010, Reid et al., 2012), focusing on compulsive use of online sexually explicit material.

All CSB subjects and comparably aged HVs were male and heterosexual given the nature of the cues. HVs were matched in a 2:1 ratio with CSB subjects to increase statistical power. Exclusionary criteria included being under 18 years of age, history of substance-use disorders, current regular user of illicit substances (including cannabis), and having a serious psychiatric disorder, including current moderate-severe major depression (Beck Depression Inventory > 20) or obsessive-compulsive disorder, or history of bipolar disorder or schizophrenia (Mini International Neuropsychiatric Inventory) (Sheehan et al., 1998). Other compulsive or behavioral addictions were exclusions, which were assessed by a psychiatrist, including problematic use of online gaming or social media, pathological gambling or compulsive shopping and binge-eating disorder.

Subjects completed the UPPS-P Impulsive Behavior Scale (Whiteside and Lynam, 2001), Beck Depression Inventory (Beck et al., 1961), State Trait Anxiety Inventory (Spielberger et al., 1983) and the Alcohol-Use Disorders Identification Test (AUDIT) (Saunders et al., 1993). The National Adult Reading Test (Nelson, 1982) was used to obtain an index of IQ.

Two CSB subjects were taking antidepressants and had comorbid generalized anxiety disorder and social phobia: social phobia (N = 1) and a childhood history of ADHD (N = 1).

Written informed consent was obtained and the study was approved by the University of Cambridge Research Ethics Committee. Subjects were paid for their participation.

### 2.2 Behavioral tasks

Twenty-two CSB subjects and 40 comparably aged male volunteers were tested in a novelty-preference task and two conditioning-preference tasks reported here, and an attentional bias task (dot-probe task) reported elsewhere (Mechelmans et al., 2014). The tasks were carried out after the fMRI experiment, in a counterbalanced order.

#### 2.2.1 Novelty preference

**Fig. 1:**
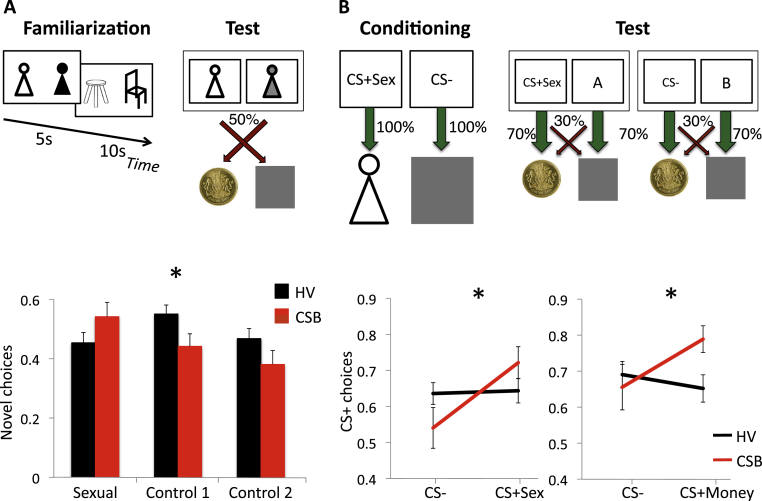
Novelty and conditioning behavioral measures. A. Novelty preference: task and outcomes. Subjects were familiarized with sexual images and two non-sexual control images followed by a choice-discrimination task involving choosing between a familiar or matched novel choice randomly (p = 0.50) associated with winning. The graph shows the proportion of novelty choices across trials in subjects with compulsive sexual behavior (CSB) and healthy volunteers (HV). B. Conditioning: task and outcomes. The sexual conditioning task is shown. During conditioning, two black-and-white visual patterns (CS+Sex and CS−) were followed by sexual or neutral images respectively. During choice discrimination testing, subjects chose between CS+Sex and CS− paired with novel visual-pattern stimuli (A and B). The CS+Sex and CS− stimuli were associated with greater probabilities of winning. The graphs show the proportion of conditioned stimuli choices across trials of CSB and HV for Sexual outcomes (left) and Monetary outcomes (right). *Group-by-Valence interaction: p < 0.05.

Subjects were familiarized to three categories of stimuli (Sexual images, Neutral human images and Neutral object images) and then performed a choice-discrimination testing phase, choosing between novel versus familiar stimuli matched within each category (Fig. 1A). In the familiarization phase, six images were shown to the participant: 2 images of undressed women (Sexual condition), 2 images of dressed women (Control1) and 2 images of a piece of furniture (Control2) (2 images per condition). The 6 images were randomly presented in pairs to the participants, in a total of 48 trials (16 trials each condition). The duration of each trial was 5 s. To ensure engagement with the task, subjects were instructed to carefully study the images because they would be asked questions during the familiarization phase. Simple questions about the images were randomly posed during the task in the inter-trial interval (e.g., to indicate which woman had her arms crossed using the right or left arrow: ‘Arms crossed’). Each question was relevant to the previously viewed pair of images, hence ensuring that subjects maintained attention to each pair of images.

In the testing phase, subjects viewed three image-pairs consisting of a familiarized image and a novel image matched for each experimental condition. Six images were used: 3 familiar, selected from the previous familiarization phase (one for each of the three conditions) and 3 new images (one novel for each condition). The image-pair was shown for 2.5 s followed by a 1-s feedback (win £1 or win nothing). A total of 60 trials (20 trials each condition) were presented. The probability of winning for any of the images was random at p = 0.50. The subject was instructed to choose one of the stimuli from the pair with the goal to make as much money as possible and told that they would receive a proportion of their earnings. They were instructed that the first trial would be a guess but that one of the stimuli would be associated with a greater likelihood of winning. The primary outcome measure was the proportion of novel choices across trials for each condition. Since the learning contingencies used here were purely random (p = 0.50), the outcome measure exclusively indicates a stimuli preference. After the study, subjects were asked to rate attractiveness of the female subjects on a scale of 1–10 following testing. Task duration was 8 min (4 min for the training and 3.5 min for the testing phase).

#### 2.2.2 Conditioning preference

Subjects were tested on two conditioning preference tasks in a counterbalanced order, both consisting of a conditioning phase and a testing phase (Fig. 1B). Both tasks had the same design but one focused on sexual and the other on monetary conditioning.

In one training phase, two visual patterns (CS+Sex, CS−), presented for 2 s, were conditioned to an image of an undressed female or a neutral grey box (1-s outcome), respectively. This was followed by an inter-trial interval of 0.5 to 1 s. Sixty trials were presented in total (30 CS^+^ and 30 CS^−^). To ensure task engagement, subjects were instructed to keep track of the number of times they saw a red square around the outcome image, and they reported this number at the end of the training phase.

The training phase was followed by a testing phase in which the CS+Sex and CS− stimuli were each paired with a novel visual-pattern stimulus (e.g. Image A or Image B respectively). Subjects were asked to choose one of the stimuli from the stimulus pair (e.g. CS+Sex or Image A; CS− or Image B; duration 2.5 s), which was followed by feedback of win £1 or win nothing (duration 1 s). The CS+Sex and CS− had a greater probability of winning (p = 0.70 win £1/p = 0.30 win nothing) relative to the novel paired stimulus (p = 0.70 win nothing/p = 0.30 win £1). Subjects were tested for 40 trials in total (20 trials per condition) and were told the goal was to make as much money as possible and that they would receive a proportion of their earnings. They were instructed that the first trial would be a guess but that one of the stimuli would be associated with a greater likelihood of winning.

In the second training and testing task, a similar task design was used paired with monetary outcomes: a different set of visual patterns was conditioned (CS+Money, CS−) to the image of £1 or a neutral grey box. Subjects were told they would win a proportion of the money they viewed. A similar testing phase followed.

As the CS+ and CS− stimuli were associated with higher probabilities of winning, we assessed novelty choice preference of the first trial to assess initial approach behaviors and the proportion of times the CS+ and CS− stimuli were selected across all trials to assess the influence of choice preference of the cue on instrumental learning. Each task lasted approximately 7 min (4 min for the training and 2.5 min for the testing phases).

### 2.3 Imaging task

Twenty CSB subjects and 20 matched HVs were scanned performing a conditioning and extinction task (Fig. 3A). In the conditioning phase, six images (colored patterns) were used as conditioned stimuli (CS+) paired with the unconditioned stimulus (US) image of an undressed female (CS+sex), £1 (CS+money) or a neutral grey box (CS−). Two CS+ were paired per outcome. Five different images of undressed females were used for the sexual outcomes and repeated 8 times over the course of conditioning. The CS+ duration was 2000 msec; at 1500 msec, the US was overlaid for 500 msec and followed by a response block with a central fixation point, which ranged from 500 to 2500 msec. To maintain attention to the task, subjects pressed the left button for the money outcome, the right button for the person outcome, and either button for the neutral outcome during the fixation period. Subjects viewed a total of 120 trials (20 per CS+ or 40 per condition) in the conditioning phase. The conditions were randomly presented. In the extinction phase, each CS+ was shown for 2000 msec without the US for a total of 90 trials (15 per CS+ or 30 per condition) followed by a fixation point (500 to 2500 msec). Thus, at 1500 msec, subjects would expect an outcome, which was unexpectedly omitted. Prior to the study, subjects were trained outside of the scanner on 20 trials of a similar design with different CS+ and images of females, money and neutral items to practice the motor response during the response block. During the practice, subjects viewed images of dressed females but were told that in the scanner, they might see explicit stimuli. All tasks were programmed using E-prime professional v2.0 software.

### 2.4 Statistical analysis of behavioral data

Subject characteristics were analyzed using independent t-tests or Chi Square. Data was inspected for outliers (>3 SD from group mean) and tested for normality of distribution (Shapiro Wilks test). Choice preference averaged across all trials for both novelty and conditioning tasks were assessed using mixed measures ANOVA with the between-subjects factor of Group (CSB, HV) and within-subject factor of Valence (Sexual, Control1, Control2; CS+, CS−). Choices for the first trial were also analyzed using Chi-Square tests. P < 0.05 was considered significant.

### 2.5 Neuroimaging

#### 2.5.1 Imaging data acquisition

Participants were scanned in a 3T Siemens Magnetom TimTrio scanner, at the Wolfson Brain Imaging Centre, University of Cambridge, with a 32-channel head coil. Anatomical images were obtained using a T1-weighted structural image using an MPRAGE sequence (TR = 2300 ms; TE = 2.98 ms; FOV 240 × 256 × 176 mm, voxel size 1 × 1 × 1 mm). fMRI data were acquired using blood oxygenation level-dependent (BOLD) contrast whole-brain echo-planar imaging (EPI) with the following parameters: 39 interleaved axial slices per volume, TR 2.32 s, TA 2.26, TE 33 ms, 3 mm slice thickness.

Data analyses were conducted using Statistical Parametric Mapping software (SPM 8) (http://www.fil.ion.ucl.ac.uk/spm). Pre-processing consisted of slice-time correction, spatial realignment, coregistration with the subjects’ T1-weighted structural images, normalization, and spatial smoothing (full-width at half-maximum of 8 mm). The first 4 volumes of each session were discarded to allow for T1-equilibration effects.

#### 2.5.2 Imaging data analysis

Statistical analyses were performed using a general-linear-model (GLM) modeling the conditioning and extinction phases for both conditioned stimuli and outcomes separately for all 3 categories. Realignment parameters were included to correct for motion artifact. The time of onset of the outcome omission in the extinction phase used was 1500 msec after the onset of the stimulus (or the time at which the outcome would have been expected in the conditioning phase) with a 500 msec duration.

For each condition, the conditioned stimuli (CS+Sex, CS+Money, CS−) were averaged across trials separately for the conditioning and extinction phase, and also for the outcome in the extinction phase. The two different stimuli were averaged within the same condition. In the second level analysis, we used a full factorial analysis (repeated measures ANOVA) comparing Group, Valence and Interactions for averaged trials. The different phases of the imaging task and description of the analyses is further illustrated in Fig. 4.

For the habituation analysis of the outcomes in the conditioning phase, we created regressors for the first and last half of the Sexual and Neutral outcomes in the first level analysis. Subjects were shown 5 different Sexual images 8 times across both CS+Sex trials. Thus for Sexual images, the first half corresponded with the first 4 Sexual image exposures for each of the 5 different images and the last half, the last 4 Sexual image exposures for each of the 5 different images. In the second level analysis, using a full factorial analysis, we compared activity in the first and last half of the Sexual versus Neutral outcomes using a between-subject factor of Group, and within-subject factors of Valence and Time. For all above analyses, whole brain cluster corrected FWE p < 0.05 was considered significant.

As we identified an interaction between Group × Valence × Time in the dACC, we then used an SPM Toolbox, MarsBaR (MARSeille Boite A Region d’Interet), to extract the beta values on a trial-by-trial basis for each individual using the dACC central coordinate and radius of 5 mm. In the first level analysis, we created regressors to evaluate change on a trial-by-trial basis. For example, 8 regressors were created for the sexual outcome consisting of different sexual outcomes shown 8 times. We calculated the slope and intercept points of each of the three outcomes for each individual. The slope and intercept points were then separately entered into a mixed-measures ANOVA comparing Group as a between-subject factor and Valence as a within-subject factor. P < 0.05 was considered significant.

Similarly, a psychophysiological-interaction analysis was conducted with the same dACC region-of-interest (ROI) seed comparing early versus late exposure to the sexual outcomes. In all analyses, activations above family-wise error (FWE) whole-brain corrected p < 0.05 and 5 contiguous voxels were considered significant. We further conducted region of interest analyses focusing on *a priori* regions using WFU PickAtlas small volume correction (SVC) FWE-corrected with a Bonferroni correction for multiple ROI comparisons (p < 0.0125).

## 3 Results

The characteristics of the CSB and HVs are reported in Table 1.

### 3.1 Behavioral results

#### 3.1.1 Novelty preference

For the averaged choice preference across 20 trials, there was a trend towards a Valence effect (F(1,59) = 2.89, p = 0.065) and a Group-by-Valence interaction (F(2,59) = 3.46, p = 0.035) and no Group effect (F(1,60) = 1.47, p = 0.230) (Fig. 1A). Given the interaction effect, we conducted post-hoc analyses, which showed that CSB subjects had greater novelty preference for Sexual versus Control2 (p = 0.039) whereas HV had greater novelty preference for Control1 versus Control2 (p = 0.024).

For the choice preference for the first trial, although CSB subjects were less likely to choose the novel compared to familiar neutral stimulus (percent of first choice novel: Sexual, Control 1, Control 2: HV: 51.6%, 58.1%, 38.7%; CSB: 50.0%, 44.4%, 22.2%) there were no significant group differences (Sexual, Control1, Control2: Chi-square = 0.012, 0.357, 0.235 p = 0.541, 0.266, 0.193).

In summary, CSB subjects were more likely to choose the novel over the familiar choice for Sexual images relative to Neutral object images whereas HVs were more likely to choose the novel choice for Neutral human female images relative to Neutral object images.

#### 3.1.2 Conditioning preference

##### 3.1.2.1 Sexual conditioning task

For the averaged choice preference over 20 trials, there was a Valence effect (F(1,60) = 5.413, p = 0.024) and a Group-by-Valence effect (F(1,60) = 4.566, p = 0.037) in which CSB subjects were more likely to select the CS+Sex versus CS− compared to HVs (Fig. 1B). There was no Group effect (F(1,60) = 0.047, p = 0.830). As there was an interaction effect, we conducted further post-hoc analyses: CSB subjects were more likely to select the CS+Sex versus the CS− (p = 0.005) but not HVs (p = 0.873). For the choice preference for the first trial, there were no differences between groups (percent of first choice CS+Sex: HV: 64.5%, CSB: 72.2%; Chi-square = 0.308, p = 0.410).

##### 3.1.2.2 Monetary conditioning task

For the averaged choice preference over 20 trials, there was no significant effect of Valence (F(1,60) = 1.450, p = 0.235) or Group (F(1,60) = 1.165, p = 0.287). There was a Group-by-Valence effect (F(1,60) = 4.761, p = 0.035) (Fig. 1B). For the choice preference for the first trial, there were no differences between groups (percent of first choice CS+Money: HV: 48.4%, CSB: 66.7%; Chi-square = 1.538 p = 0.173).

CSB subjects (attractiveness score 8.35, SD 1.49) had similar ratings of attractiveness of all female images relative to HVs (8.13, SD 1.45; t = 0.566, p = 0.573).

Thus, CSB subjects had greater preference for stimuli conditioned to either sexual images or money.

#### 3.1.3 Relationship between choice preferences and attentional bias

**Fig. 2:**
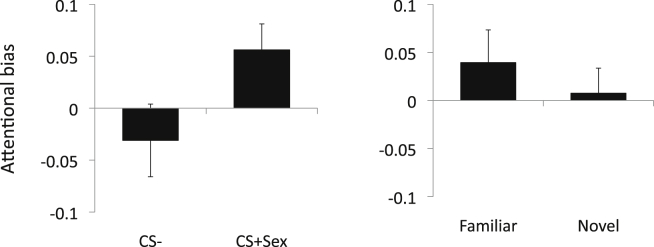
Relationship between choice preferences and attentional bias across groups. The left graph shows early attentional bias scores for sexual versus neutral stimuli (higher scores indicated greater bias towards sexual versus neutral stimuli) in subjects who preferred the CS+Sex as compared to CS− as the first choice across both groups. *p < 0.05. The right graph shows early attentional bias scores for sexual versus neutral stimuli in subjects who preferred the novel sexual stimulus as compared to the familiar stimulus.

We further investigated if there was any relationship between our previously published findings of enhanced attentional bias to sexual images (Mechelmans et al., 2014) and the current findings of initial choice preference for novelty or for CS+Sex. Using independent t-tests we assessed early attentional bias for the sexual versus neutral images comparing choice preference for subjects who chose the CS− versus CS+Sex and separately Familiar versus Novel stimuli. Across both groups, subjects who chose the CS+Sex as compared to those who chose the CS− had enhanced attentional bias for the sexual versus neutral stimuli (t = −2.05, p = 0.044). In contrast, there was no statistically significant difference between subjects who chose the Novel as compared to the Familiar and attentional bias scores for the sexual compared to neutral stimuli (t = 0.751, p = 0.458) (Fig. 2).

Thus, our previously reported findings of early attentional bias may be related to conditioning preferences for sexual stimuli rather than novelty preferences for sexual stimuli.

### 3.2 Imaging results

#### 3.2.1 Conditioning: cue

We first assessed averaged cue-conditioning across all trials. There was no Group effect. There was a Valence effect in which exposure to conditioned stimuli to Money (CS+Mon) and Sex (CS+Sex) as compared to Neutral (CS−) stimuli was associated with greater activity in the occipital cortex (all the following p-values report whole brain cluster corrected FWE p < 0.05: peak cluster in Montreal Neurological Institute coordinates: X Y Z in mm: −6 −88 −6, Cluster size = 3948, whole brain FWE p < 0.0001), left primary motor cortex (X Y Z = −34 −24 52, Cluster size = 5518, whole brain FWE p < 0.0001) and bilateral putamen (left: X Y Z = −24 −2 4, Cluster size = 338, whole brain FWE p < 0.0001; right:X Y Z = 24 4 2, Cluster size = 448, FWE p < 0.0001), and thalamus (X Y Z = −0 −22 0, Cluster size = 797, p < 0.0001) activity. There was no Group-by-Valence interaction.

#### 3.2.2 Extinction: cue

We then assessed the Extinction phase of the conditioned stimuli. There was a Valence effect in which CS+Sex and CS+Mon versus CS− exposure was associated with greater occipital cortex activity (X Y Z = −10 −94 2, Cluster size = 2172, whole brain FWE p < 0.0001). There were no Group or Interaction effects.

#### 3.2.3 Acquisition: outcome

**Fig. 3:**
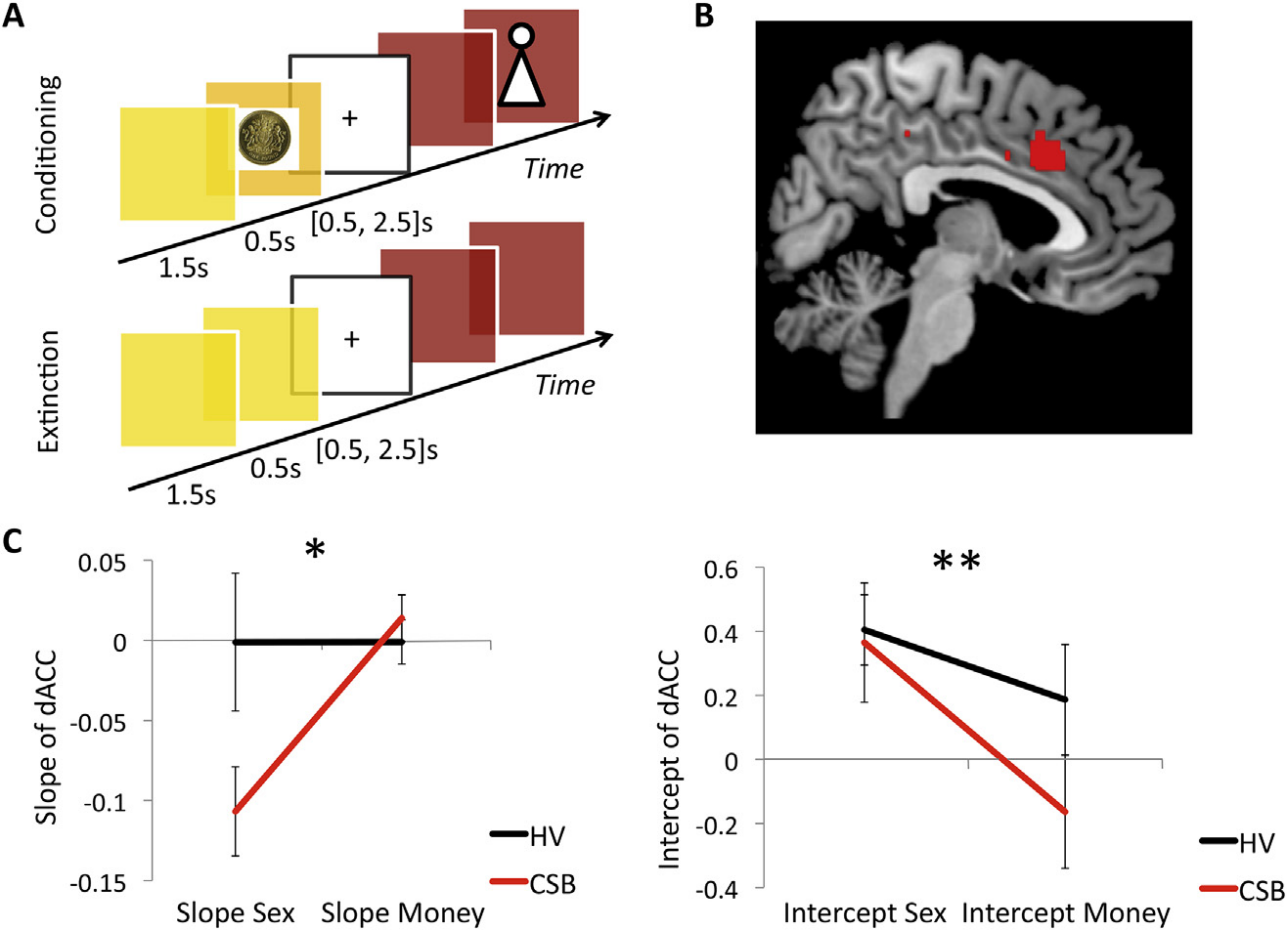
Conditioning imaging task and habituation. A. Imaging task. During conditioning, subjects viewed six colored patterns followed by a Sexual, Monetary or Neutral image. The extinction phase followed, during which the conditioned stimulus was shown without the unconditioned stimulus. B. Habituation. Habituation of dorsal anterior cingulate (dACC) activity in compulsive sexual behavior (CSB) subjects versus healthy volunteers (HV) to repeated Sexual versus Neutral images. The image shows the comparison of the first and last half of the trials. C. Slope and intercept of dACC habituation. The graphs show the slope or degree of habituation (left graph) of beta values of the dACC in CSB and HV individuals and the intercept or initial activity of CSB versus HV (right graph) of Sexual – Neutral (Sex) and Monetary – Neutral (Money) images. *Valence and Group-by-Valence effects p < 0.05; **Valence effect p < 0.05.

**Fig. 4:**
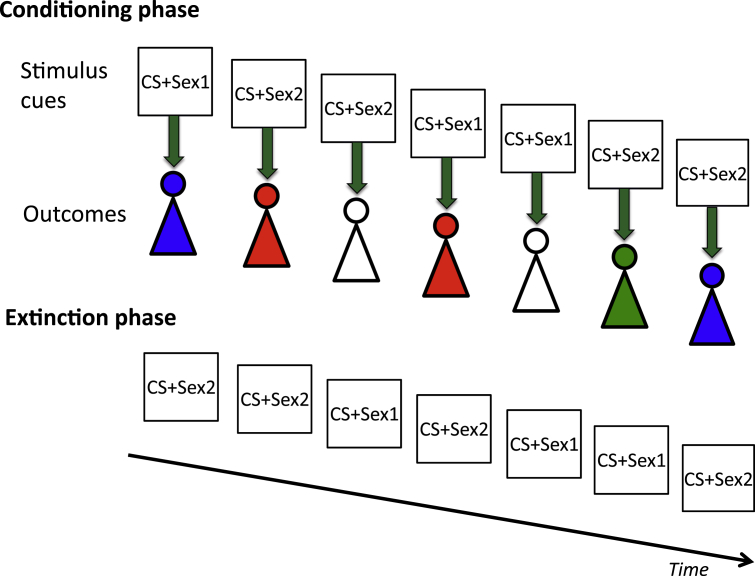
Illustration of conditioning, habituation and extinction. This figure illustrates the phases of the imaging task in which the conditioned stimuli are paired with outcomes (CS+sex shown here; CS+money conditioned to monetary outcomes and CS− conditioned to neutral outcomes were randomly interspersed and are not shown) and extinction phase in which only the conditioned stimuli are shown without the outcome. Two different CS+ for each outcome type or CS− were conditioned over 20 trials per stimulus. Five different sexual images (shown here with differing colours of the female stick image) were randomly paired with the two different CS+sex and were each shown 8 times. For the habituation analysis, the change in time of these repeated outcomes was analyzed. (For interpretation of the references to colour in this figure legend, the reader is referred to the web version of this article.)

To examine effects of habituation to sexual novelty, we first investigated if any regions had a greater decrease in activity to Sexual outcomes in CSB subjects compared to HVs by comparing the Group × Valence × Time interaction of the first and last half of the Sexual imagery versus Neutral outcome phase. CSB subjects had greater decrease in dorsal anterior cingulate cortex (dACC) activity over time (X Y Z = 0 18 36, Cluster size = 391, whole brain FWE p = 0.02) and right inferior temporal cortex (X Y Z = 54 −36 −4, Cluster size = 184, whole brain FWE p = 0.04) to Sexual versus Neutral outcomes compared to HVs (Fig. 3B).

We then extracted the trial-by-trial beta values focusing on the dACC for Sexual, Monetary and Neutral outcomes. We compared the slopes (i.e., degree of habituation) and intercept points (i.e., activity to initial exposure) comparing Sexual – Neutral and Monetary – Neutral outcomes (Fig. 3C). For the slope, there was a main effect of Valence (F(1,36) = 6.310, p = 0.017) and a Group-by-Valence interaction (F(1,36) = 6.288, p = 0.017). As there was an interaction effect, we conducted post-hoc analyses: there was a steeper decrease in dACC slope to Sexual outcomes in CSB compared to HVs (F = 4.159, p = 0.049) with no differences to Monetary outcomes (F = 0.552, p = 0.463). There was no main effect of Group (F(1,36) = 2.135, p = 0.153). For the intercept value, there was a main effect of Valence (F(1,36) = 11.527, p = 0.002) but no main effect of Group (F(1,36) = 0.913, p = 0.346) or interaction effect (F(1,36) = 2.067, p = 0.159). There were no correlations between the conditioning and outcome phases.

#### 3.2.4 Extinction: outcome

**Fig. 5:**
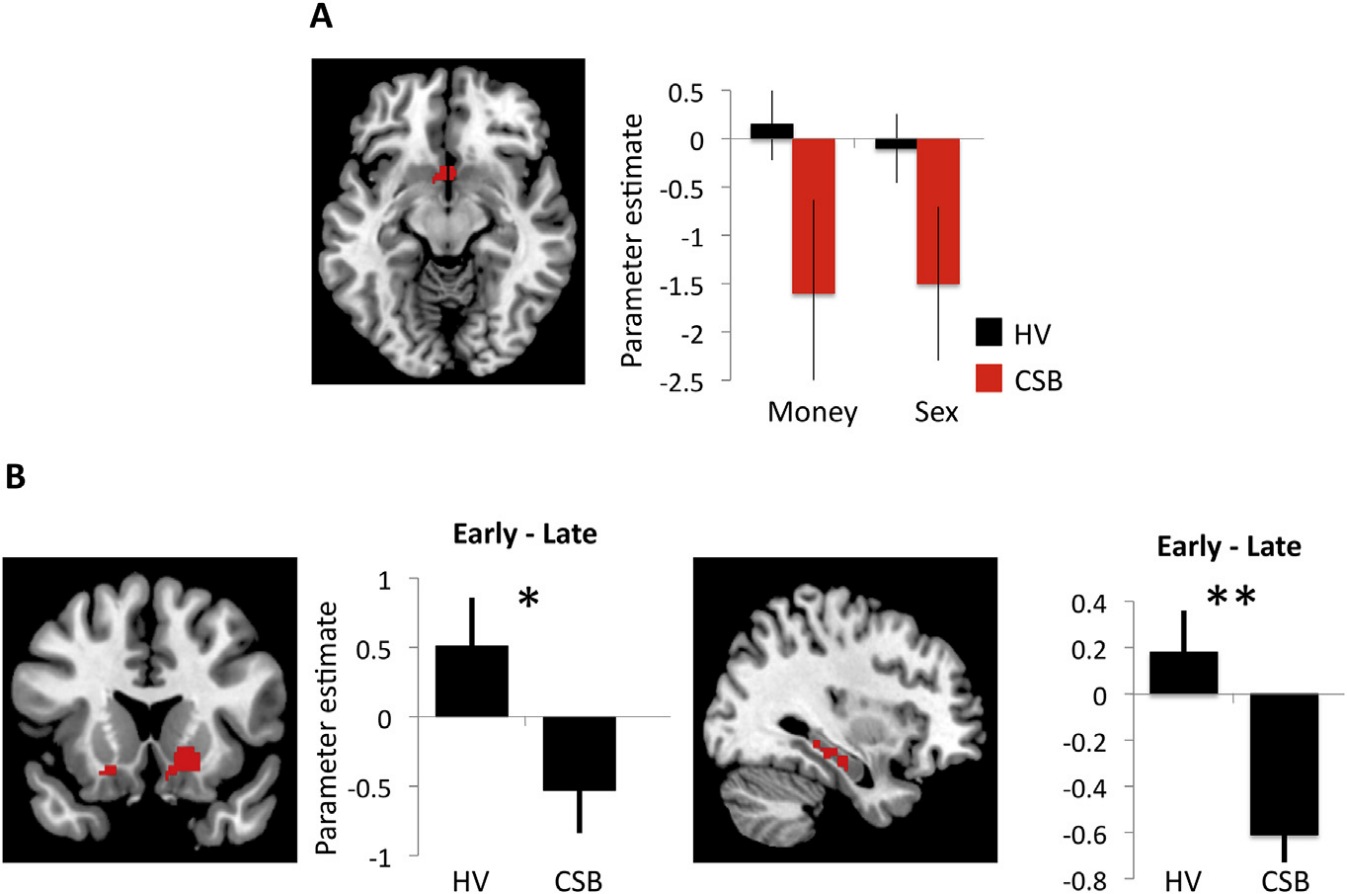
Extinction and functional connectivity. A. Omission of outcome during extinction. Decreased right ventral striatal activity in both groups for unexpected omission of Sexual and Monetary outcomes versus Neutral outcomes during extinction (Valence effect: p < 0.05). B. Functional connectivity with repeated exposure. Psychophysiological interaction of individuals with compulsive sexual behaviours (CSB) and healthy volunteers (HV) individuals comparing early versus late exposure of sexual outcomes with a dorsal cingulate seed showing functional connectivity with right ventral striatum (left) and bilateral hippocampus (right). *p < 0.05; **p < 0.005.

We assessed the omission of outcome during the Extinction phase across all trials. Here we had a very specific prediction that ventral striatal activity was decreased during outcome omission to previously rewarding outcomes consistent with a negative prediction error. There was an effect of Valence in which lower right ventral striatal activity was observed to the lack of Sexual and Monetary outcomes compared to Neutral outcomes (X Y Z 2 8–10, Z = 3.59, SVC FWE corrected p = 0.036) (Fig. 5A). There were no Group or interaction effects. There were no significant differences between Sexual and Monetary outcomes.

### 3.3 Functional connectivity of dorsal cingulate

Functional connectivity using psychophysiological interaction of the dACC contrasting early versus late exposure (the first 2 trials versus the last 2 trials) of the Sexual outcomes was also assessed. There was greater functional connectivity in the HVs compared to CSB subjects in the early compared to late trials between the dACC and right ventral striatum (X Y Z = 18 20 −8 mm, Z = 3.11, SVC FWE-corrected p = 0.027) and bilateral hippocampus (right: X Y Z = 32–34 −8, Z = 3.68, SVC FWE-corrected p = 0.003; left: X Y Z = −26 −38 04, Z = 3.65 SVC FWE-corrected p = 0.003) (Fig. 5B). Thus CSB subjects had greater functional connectivity between these regions late in exposure whereas healthy volunteers had greater functional connectivity early in exposure.

### 3.4 Relationship between behavioral and imaging results

We investigated if there was a relationship between dACC habituation (slope) of the Sexual outcome with novelty preference for Sex – Control2 using Pearson correlation. Across subjects, the novelty preference for Sexual versus Control2 images was negatively correlated with the slope for sexual images (r = −0.404, p = 0.037). Thus, greater sexual novelty preference was correlated with a more negative slope or greater dACC habituation.

## 4 Discussion

We show that CSB individuals had greater choice preference for novel sexual images and for cues conditioned to both sexual and monetary stimuli compared to healthy volunteers. CSB subjects also had greater habituation of dACC activity to repeated sexual versus monetary images. Across all subjects, the degree of dACC habituation to sexual stimuli was associated with greater novelty preference for sexual images. This study builds on our previous findings of enhanced attentional bias (Mechelmans et al., 2014) and cue reactivity (Voon et al., 2014) towards explicit sexual cues in CSB implicating a dACC-(ventral striatal)-amygdalar network. Here, we show that early attentional bias to sexual cues assessed using a dot-probe task was associated with greater approach behaviors towards cues conditioned to sexual images but not novelty preference. Thus, the findings indicate that possible mechanisms underlying early attentional bias to sexual cues observed in CSB subjects are closely aligned with cue-conditioning and enhanced approach behaviors towards sexual conditioned cues. Although novelty preference to sexual stimuli is also enhanced in CSB subjects, this behavior is unrelated to the observation of early attentional bias. This observation contrasts with a previous study in healthy volunteers, which shows a relation between attentional bias towards sexual stimuli and sexual sensation-seeking (Kagerer et al., 2014). This may be explained by a greater influence of cue-conditioning in individuals with pathology.

### 4.1 Preference for stimuli conditioned to sexual or monetary rewards

This enhanced preference for conditioned stimuli across both forms of reward (sexual and monetary rewards) suggests either that CSB subjects have greater reward sensitivity or generalization and transfer of the effects of conditioning between similar stimuli (Mazur, 2002). This phenomenon is in line with behavioral cross-sensitization observed in rodent studies between stimulants and incentive properties of natural rewards, such as sex proposed to involve dopaminergic mechanisms (Fiorino and Phillips, 1999, Frohmader et al., 2011). Applying such investigative approaches to individuals with other non-substance addictions like gambling disorder is warranted as initial studies have suggested differential neural activation patterns to monetary and sexual rewards in this population (Sescousse et al., 2013).

Although we have used the term habituation to explain the decrease in activity to repeated sexual stimuli, as this is assessed in the context of cue-conditioning during which cues are paired with the outcomes, one relevant process may be the effect of associative learning underlying the cue-conditioning in which dopaminergic activity to the unexpected reward shifts towards the cue with conditioning and thus decreases over time such that activity as the reward outcome becomes expected will decrease over time (Schultz, 1998). However, as (i) we randomized the 5 sexual images repeated 8 times across the two stimuli conditioned to sexual rewards; (ii) we did not observe any relationship between the decrease in dACC activity to repeated sexual stimuli with conditioning preference but did observe a relationship with sexual novelty preference, (iii) there were no group differences in imaging outcomes to the conditioned cues and no evidence of enhanced conditioning specific to sexual rewards, and (iv) CSB subjects had a preference for both stimuli conditioned to sexual and monetary rewards, we have suggested the process may be consistent with a habituation effect.

We further show that the unexpected lack of sexual or monetary reward is associated with lower right ventral–striatal activity across all subjects. Converging primate and human studies suggest that phasic dopamine encodes a prediction error with a positive prediction error to unexpected reward and a negative prediction error to unexpected lack of reward (Pessiglione et al., 2006, Schultz, 1998). This decrease in ventral–striatal activity to the unexpected lack of sexual or monetary rewards may be consistent with a negative prediction error, suggesting similar mechanisms underlying secondary and primary rewards, both of which may elicit conditioned preferences.

### 4.2 Preference for novel sexual stimuli and dorsal cingulate habituation

Novelty-seeking and sensation-seeking are associated with disorders of addiction across a range of substances including tobacco, alcohol and drug use (Djamshidian et al., 2011, Kreek et al., 2005, Wills et al., 1994). Preclinical studies demonstrate a role for novelty preference as a risk factor for drug-seeking behaviors (Beckmann et al., 2011, Belin et al., 2011), and similarly, higher sensation-seeking is a predictor of subsequent binge drinking in adolescents but not of eating disorders (Conrod et al., 2013). Likewise, in Parkinson's patients who develop impulse control behaviors on dopamine agonists, novelty seeking is associated with externalizing rewards such as pathological gambling and compulsive shopping but not natural rewards such as binge eating or CSB (Voon et al., 2011). In our current study, there were no differences in sensation-seeking scores between CSB subjects and HVs, suggesting a role for novelty preference specific to the reward but not generalized novelty- or sensation-seeking. Our findings may be particularly relevant in the context of online explicit stimuli, which potentially provides an endless source of novelty, and may indeed differ from drug addiction in which ongoing novelty may be less of an issue.

We further show that CSB subjects had more rapid habituation of the dACC to repeated sexual images relative to monetary images. This finding may reflect repeated exposure to explicit online stimuli, similar to the observation of decreased putaminal activity to excessive use of online explicit materials in healthy male volunteers (Kuhn and Gallinat, 2014). Across all subjects, novelty preference to repeated sexual images was predicted by a greater habituation of dACC activity to sexual outcomes. We have recently shown enhanced dACC activity in CSB subjects to explicit videos (Voon et al., 2014), and the dACC has been implicated in both drug-cue reactivity and craving (Kuhn and Gallinat, 2011). In this previous study, the videos were sexually explicit and may have acted as conditioned cues and were shown infrequently, and hence they may have been less likely to be associated with habituation. Habituation was also not specifically assessed. The dACC receives extensive projections from midbrain dopaminergic neurons and is well-localized with multiple cortical connections to influence action selection. The dACC plays a role in detecting and planning appropriate behavioral responses to salient events during continuous behavioral adaptation (Sheth et al., 2012). Alternatively, the dACC is also implicated in reward-motivated behaviors, particularly prediction about future rewards and reward-prediction errors (Bush et al., 2002, Rushworth and Behrens, 2008). Thus, the role of the dACC may be related to effects of salience or unexpected reward.

The assessment of novelty involves the comparison of incoming information with stored memory mediated by a polysynaptic hippocampal-(ventral striatal)-(ventral tegmental area) loop suggested to combine information on novelty, salience and goals (Lisman and Grace, 2005). Our observation of enhanced dACC-(ventral striatal)-hippocampal connectivity in CSB subjects with repeated exposure to sexual outcomes despite a decrease in dACC activity may represent a network involved in the aberrant encoding of hippocampal-dependent memory to repeated sexual imagery.

The study has important strengths. This is the first investigation into the neural underpinnings of novelty and cue-conditioning processes in CSB, with the investigation permitting insights into specific aspects of the behavioral and neural correlates of these processes. We show experimentally what is observed clinically that CSB is characterized by novelty-seeking, conditioning and habituation to sexual stimuli in males. However, some limitations should also be acknowledged. First, the study involved solely young heterosexual men. Although this feature can be seen as a strength by limiting heterogeneity, it can also be a limitation with respect to generalization to women, other age groups and individuals with other sexual orientations. Second, CSB participants were generally more anxious, depressed, and impulsive and showed a trend for more obsessive-compulsive features. Although we did not find a direct effect of these variables in our results, we cannot exclude the possibility that they may have influenced the findings. Third, there were no significant differences in the imaging analyses of conditioning, extinction cues, extinction outcome. Our imaging findings support the behavioral processes of sexual novelty but we did not observe imaging findings to support the findings of conditioning preferences. Larger samples, more explicit images, or facilitating consolidation with subsequent testing represent important considerations for future studies that may generate different results. Fourth, this study used imagery that could be perceived as erotic rather than sexually explicit. Further studies using sexually explicit materials may differentiate between conditioning effects to monetary and sexually explicit stimuli.

We highlight the role of enhanced preference for sexual novelty and a generalized enhancement of conditioning to rewards in CSB subjects involving dACC habituation. These findings extend our recent observations that CSB subjects have greater sexual cue reactivity in a network involving the dACC, ventral striatum and amygdala (Voon et al., 2014) and enhanced attentional bias to sexually explicit cues (Mechelmans et al., 2014). We emphasize a role for cue-conditioning dissociable from novelty preference underlying this observation of enhanced early attentional bias for sexual cues. These findings have potential wider relevance as the Internet provides a vast source of novel and potentially rewarding stimuli, particularly with respect to sexually explicit material. Future studies should examine the extent to which the current findings might relate to clinically relevant measures related to CSB, both cross-sectionally and prospectively. These findings suggest a role for targeting dissociable cognitive processes in the therapeutic management of CSB.

## Conflicts of interest

The material is original research, has not been previously published and has not been submitted for publication elsewhere. Authors PB, LM, SM, NH, MNP and VV declare no competing financial interests.

## Role of funding source

PB is supported by the Portuguese Foundation for Science and Technology (individual fellowship: SFRH/BD/33889/2009). Dr. Voon is a Wellcome Trust Intermediate Fellow and the study was funded by the Wellcome Trust (WT093705/Z/10/Z). Channel 4 was involved in assisting with recruitment by placing ethics-approved advertisements for the study on Internet sites. The advertisements provided contact details of the study researchers for interested participants.

## Author contributions

Conceived and designed the experiments: VV. Performed the experiments: PB, SM and VV. Analyzed the data: PB, LSM, SM, VV. Wrote the paper: PB, NAH, MNP and VV.

## Figures and Tables

**Table 1:** Subject characteristics.

		CSB	HV	T/Chi square	P
Number		22	40		
Age		25.14 (4.68)	25.20 (6.62)	0.037	0.970
Abstinence (days)		32 (28.41)			
Education	High school	22	40	0.000	1.000
Current Univ.	6	13	0.182	0.777
College degree	3	5	0.039	1.000
Univ. undergrad	9	14	0.212	0.784
Masters degree	6	3	4.472	0.057
IQ		110.49 (5.83)	111.29 (8.39)	0.397	0.692
Relationship status	Single	10	16	0.173	0.790
Curr. relationship	7	16	0.407	0.591
Married	5	8	0.064	1.000
Occupation	Student	7	15	0.200	0.784
Part-time work	3	2	1.428	0.337
Full-time work	12	21	0.024	1.000
Unemployed	0	2	1.137	0.535
Medications	Antidepressants	2			
Current Smoking status	Smokers	0	1		
Body mass index		24.91 (3.64)	23.19 (4.38)	1.566	0.122
Binge Eating	BES	6.91 (6.46)	5.72 (6.17)	0.715	0.478
Alcohol use	AUDIT	7.13 (4.11)	6.29 (3.41)	0.862	0.392
Depression	BDI	11.03 (9.81)	5.38 (4.89)	3.039	0.004
Anxiety	SSAI	44.59 (13.19)	36.15 (13.29)	2.370	0.021
STAI	49.54 (13.91)	38.23 (14.57)	2.971	0.004
Obsessive compulsive	OCI-R	19.23 (17.38)	12.29 (11.72)	1.872	0.067
Impulsivity	UPPS-P	150.83 (17.95)	130.26 (23.49)	3.569	<0.001

Abbreviations: CSB = subjects with compulsive sexual behavior; HV = healthy volunteers; BES = Binge Eating Scale; AUDIT = Alcohol Use Disorders Identification Test; BDI = Beck Depression Inventory; SSAI/STAI = Speilberger State and Trait Anxiety Inventory; OCI-R = Obsessive Compulsive Inventory; UPPS-P = UPPS Impulsive Behavior Scale.
